# BMAL1-dependent regulation of the mTOR signaling pathway delays aging

**DOI:** 10.18632/aging.100633

**Published:** 2014-01-29

**Authors:** Rohini V. Khapre, Anna A. Kondratova, Sonal Patel, Yuliya Dubrovsky, Michelle Wrobel, Marina P. Antoch, Roman V. Kondratov

**Affiliations:** ^1^ Center for Gene Regulation in Health and Diseases, BGES, Cleveland State University, Cleveland, OH; ^2^ Department of Molecular Genetics, Cleveland Clinic, Cleveland, OH; ^3^ Tartis Aging, Inc, 640 Ellicott Street, Ste. 444, Buffalo, NY 14203-; ^4^ Department of Molecular and Cellular Biology, Roswell Park Cancer Institute, Buffalo, NY 14263

**Keywords:** biological clock, metabolism, aging, cell signaling, cell growth, proliferation

## Abstract

The circadian clock, an internal time-keeping system, has been linked with control of aging, but molecular mechanisms of regulation are not known. BMAL1 is a transcriptional factor and core component of the circadian clock; BMAL1 deficiency is associated with premature aging and reduced lifespan. Here we report that activity of mammalian Target of Rapamycin Complex 1 (mTORC1) is increased upon BMAL1 deficiency both *in vivo* and in cell culture. Increased mTOR signaling is associated with accelerated aging; in accordance with that, treatment with the mTORC1 inhibitor rapamycin increased lifespan of *Bmal1−/−* mice by 50%. Our data suggest that BMAL1 is a negative regulator of mTORC1 signaling. We propose that the circadian clock controls the activity of the mTOR pathway through BMAL1-dependent mechanisms and this regulation is important for control of aging and metabolism.

## INTRODUCTION

The Target of Rapamycin (TOR) signaling pathway is a critical regulator of anabolic activities, growth and proliferation of cells [1, 2]. The role of the TOR pathway in aging is evolutionary conserved: increased TOR activity leads to accelerated aging, while reduction of TOR signaling results in increased longevity in different organisms [3, 4] including mammals. In humans, deregulated mTOR (the mammalian TOR) signaling is associated with cancer and metabolic diseases [5]. Little is known about physiological systems that are involved in regulation of TOR pathway *in vivo*.

The circadian clock, an internal time-keeping system, regulates physiological processes through generation of circadian rhythms in gene expression, which are translated into rhythms in metabolism and behavior [6-8]. Disruption of the circadian clock has been observed in many pathological conditions, and is considered to contribute to metabolic syndromes, cardiovascular diseases and cancer [9-12]. The circadian clock is also implicated in control of aging [11]: mice deficient for the core clock protein BMAL1, which develop severe premature aging phenotype [13], are the most striking example of this. The circadian clock controls glucose and lipid homeostasis [14], cell redox state regulation [15], cell cycle [16] and genotoxic stress response [17]. All these signaling pathways contribute to the circadian control of metabolism. Both clock-dependent regulation of reactive oxygen species homeostasis [18] and circadian control of the activity of histone deacetylases from the sirtuin family [19, 20] were proposed as potential molecular links between the circadian clock and aging [21]. Interestingly, circadian de-regulation is considered as a risk factor for a similar spectrum of diseases as disruption of the mTOR pathway.

Here we investigated the involvement of the circadian clock in regulation of the mTOR signaling pathway in mammals. Using a variety of *in vitro* and *in vivo* approaches we show that the circadian and mTOR pathways are connected through the activity of BMAL1 and this connection is important for delay of aging.

## RESULTS

### BMAL1 inhibits activity of mTORC1 in cells

In order to study the mTOR signaling pathway as a potential molecular mechanism of BMAL1-dependent control of aging, we decided to compare activity of mTOR signaling in cells isolated from wild type and BMAL1-deficient mice. mTOR is a serine/threonine protein kinase found in two complexes: mTOR Complex 1 (mTORC1) and mTOR Complex 2 (mTORC2). mTORC1 activity is implicated in regulation of metabolism and aging, while the role of mTORC2 in these processes is considered to be less significant [5], therefore, we assayed the activity of mTORC1. mTORC1 phosphorylates a number of downstream targets including ribosomal protein S6 kinase 1 (S6K1) and elongation factor 4E binding proteins (4EBPs) [2, 22-25]. S6K1 is phosphorylated by mTORC1 on threonine 389 (T389); 4EBP1 is phosphorylated on threonine 37 and 46 (T37/T46). S6K1, in turn, phosphorylates its target - ribosomal protein S6 - on serines 240 and 244 (Ser240/Ser244) and serines 235 and 236 (Ser235/Ser236) [25-27]. Thus, although S6 is not a direct target of mTORC1, phosphorylation of S6 is often used as an indicator of mTORC1 activity.

**Figure 1:**
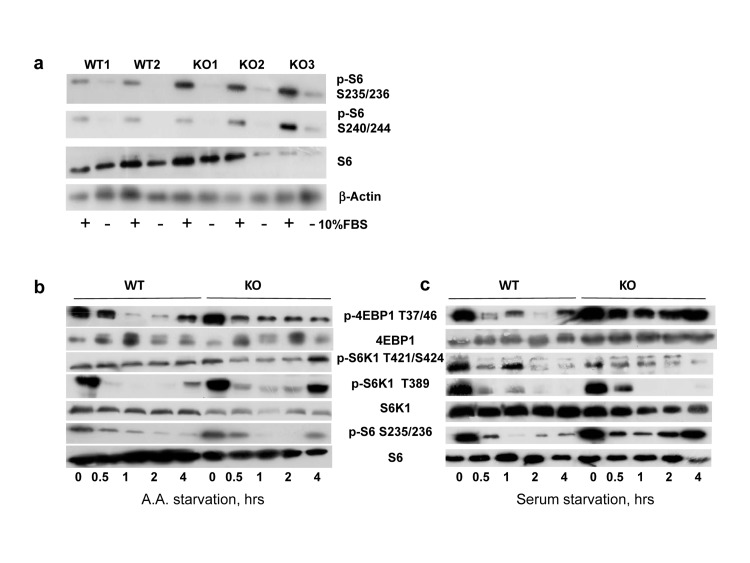
BMAL1 is negative regulator of TORC1 activity in cells **(a)** Phosphorylation and total protein level of ribosomal S6 protein in primary lung fibroblasts isolated from wild type and *Bmal1^−/−^* mice. Protein phosphorylation in cellular extracts were assayed by western blotting procedure with antibodies recognizing the indicated proteins or protein modifications WT1, WT2, KO1, KO2 and KO3 represent independently isolated populations fibroblasts isolated from wild type (WT) and *Bmal1^−/−^* (KO) mice. Cells were incubated in DMEM with 10% FBS (+) or serum deprived for 24 hrs (−). **(b)** and **(c)** Wild type (WT) and *Bmal1^−/−^* (KO) fibroblasts were subject to amino acids **(b)** or serum **(c)** withdrawal for indicated period of time. Kinetics of changes in phosphorylation of mTORC1 downstream targets are shown on the representative western blotting.

Figure 1a shows results obtained in several independently isolated populations of lung fibroblasts; phosphorylation of S6 protein was significantly higher in *Bmal1^−/−^* cells, suggesting that BMAL1 is a negative regulator of mTOR signaling. Since mTORC1 activity was shown to be sensitive to growth factor and amino acid withdrawal [28-30], to investigate the role of BMAL1 in these processes we subjected wild type and *Bmal1−/−* cells for serum or amino acid starvation. As expected, amino acid (Figure 1b) or serum (Figure 1c) starvation resulted in suppression of phosphorylation of the mTORC1 targets in both cell types. However, the level of phosphorylation and kinetics of response to starvation displayed prominent differences. When compared to wild type, *Bmal1^−/−^* cells demonstrated increased phosphorylation for mTORC1 targets (S6K1 T389 site and 4EBP-1 T37/46 sites), but showed no difference in S6K1 phosphorylation on the MAPK-specific Thr421/Ser424 site, suggesting that the regulation of mTORC1 activity by BMAL1 is highly specific.

**Figure 2:**
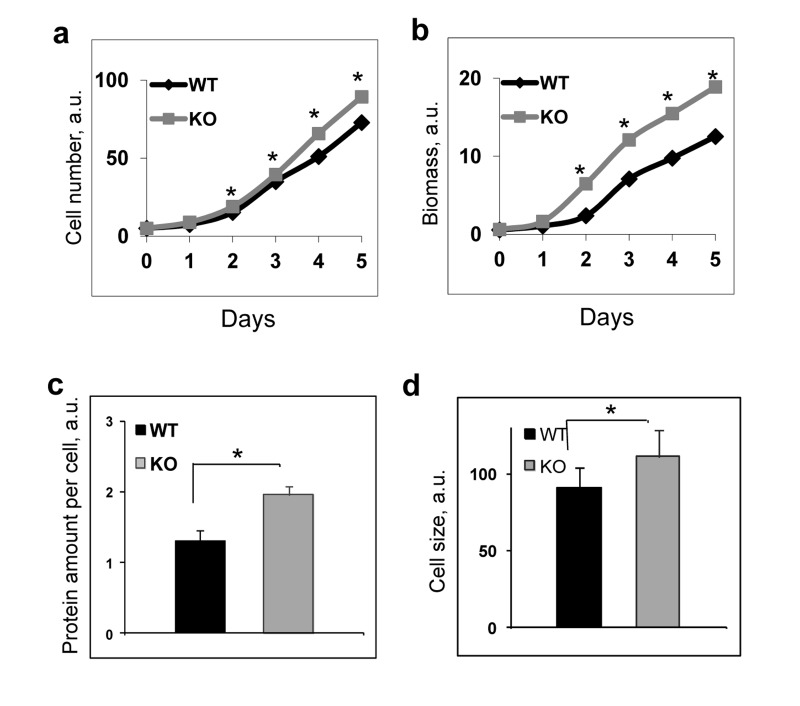
BMAL1 deficiency increases cell growth and proliferation To assay proliferation wild type and *Bmal1^−/−^* cells were plated at equal density on day 0, every 24 hrs cells were collected and counted (cell number), in parallel set of experiments the plates were stained with crystal violet reagent and biomass accumulation was estimated based on the amount of extracted dye. Results represent average for 4 plates for every time point for both genotypes. Experiments were repeated three times independently with similar results. **(a)** and **(b)** Proliferations of wild type (black diamonds) and *Bmal1^−/−^* (grey squares) fibroblasts in cell culture were assayed by counting cell number **(a)** or by measuring biomass accumulation **(b). (c)** Protein amount per cell for wild type (black bar) and *Bmal1^−/−^* (grey bar) fibroblasts. **(d)** Cell size for wild type (black bar) and *Bmal1^−/−^* (grey bars) fibroblasts were assayed by using FACSalibor cell sorter (Becton-Dikinson) and appropriate software.

It is known that the increased mTORC1 signaling is associated with increased biosynthesis and cell growth [5]. We assayed proliferation of wild type and *Bmal1^−/−^* fibroblasts and found that *Bmal1^−/−^* cells grew faster than wild type: both the cell number (Figure 2a) and especially accumulated biomass (Figure 2b) demonstrated significant increase. In agreement with that, we detected increased protein content per cell and increased cell size for *Bmal1^−/−^* fibroblasts in comparison with wild type fibroblasts (Figure 2c and 2d). These data indicate that BMAL1 acts as a negative regulator of the mTORC1 signaling pathway.

### Deficiency of Bmal1 leads to increased mTORC1 activity in vivo

**Figure 3:**
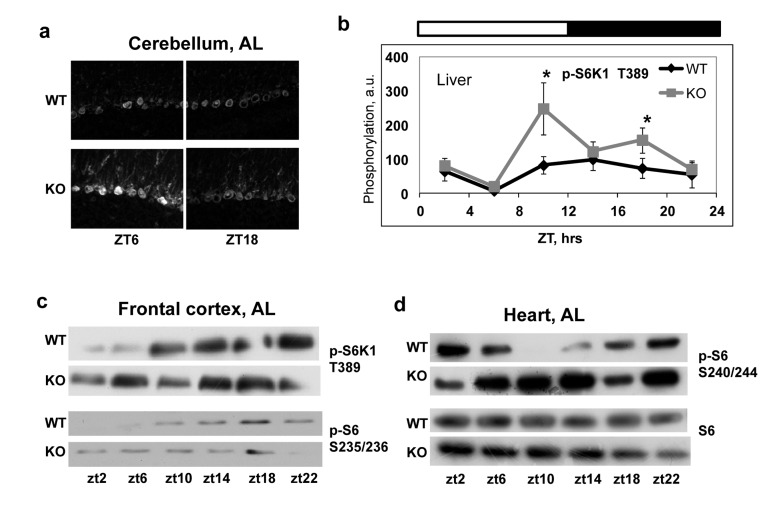
Increased mTORC1 signaling in tissues of *Bmal1^−/−^* mice Activity of TORC1 was compared in the different tissues of wild type and *Bmal1^−/−^* mice. Protein phosphorylation in tissue extracts were analyzed by western blotting procedure with antibodies recognizing the indicated proteins or protein modifications or by in situ staining on 10μM frozen tissue sections. **(a)** In situ staining of cerebellum for p-S6 S235/236. **(b)** Phosphorylation of S6K1(T389) in the liver through the circadian cycle (wild type (black diamonds) and *Bmal1^−/−^* (grey squares)). **(c)** and **(d)** Representative WB of mTORC1 downstream targets phosphorylation in the frontal cortex **(c)** and in the heart **(d)**. Bars on the top of the figure represent light (open bars) and dark (black bars) parts of the day. Zt is an abbreviation for Zeitgeber Time, zt0 is the time when light is on, the time when light is off is zt12, thus, for example zt2 represents 2hrs after light is on. * - statistically significant difference between the genotypes

**Figure 4:**
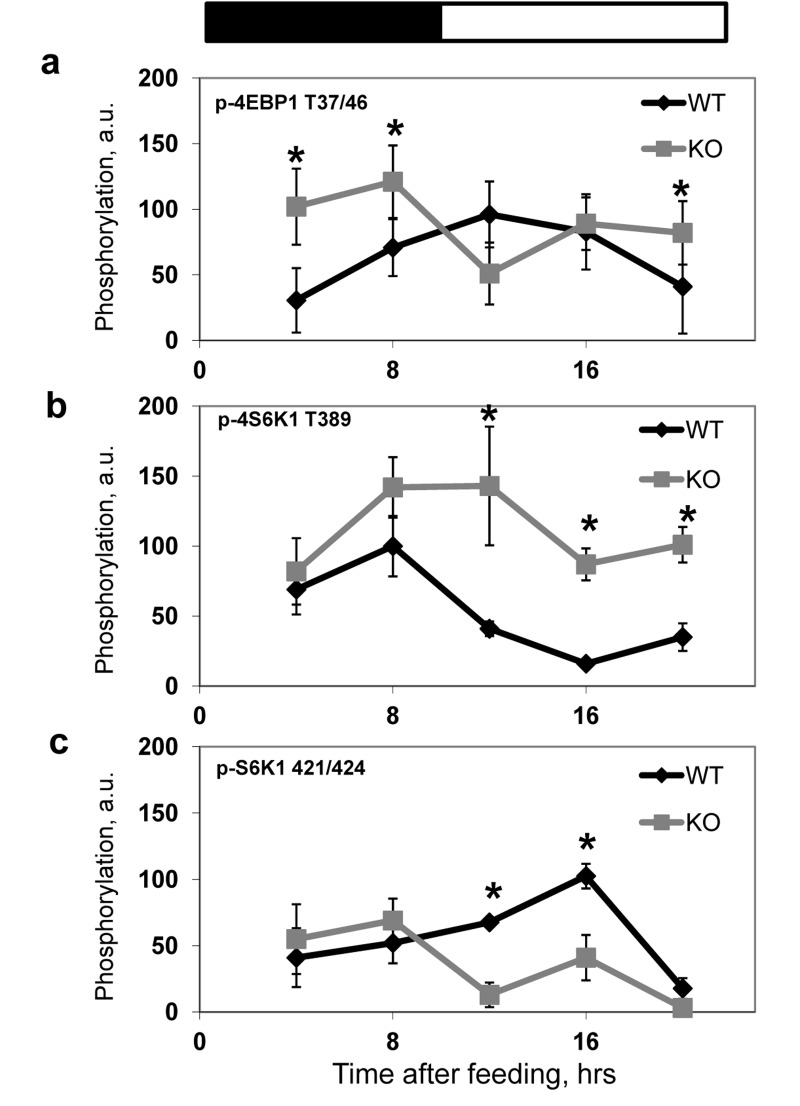
BMAL1 deficiency results in increased mTORC1 signaling in the liver of TRF mice Phosphorylation and expression of TORC1 downstream targets were analyzed by western blotting, image analyzing software was used to quantify the intensity of bands. For every experiment the value of maximum intensity of the band for wild type mice was set as 100, the intensity of bands for other time points and for *Bmal1^−/−^* mice was normalized accordingly. Results represent average for 4 animals of each genotype for each time point. **(a-c)** Quantitative profiles of TORC1 downstream targets phosphorylation in the liver of wild type (black diamonds) and *Bmal1^−/−^* (grey squares) TRF mice. **(a)** 4E-BP1 T37/46 phosphorylation. **(b)** S6K1 T389 phosphorylation; **(c)** S6K1 T421/424 phosphorylation. * - statistically significant difference between the genotypes. Food was provided at time point 0. Bars on the top of the figure represent light (open bars) and dark (black bars) parts of the day.

In order to investigate if BMAL1 is involved in the regulation of mTORC1 activity *in vivo*, we compared temporal profiles of phosphorylation of the mTORC1 targets in tissues of wild type and *Bmal1^−/−^* mice. We assayed the activity of mTORC1 in the liver (Figure 3b), heart (Figure 3d) and several brain regions (cerebellum, Figure 3a and frontal cortex, Figure 3c) isolated from wild type and *Bmal1^−/−^* mice across the circadian cycle (on Figure 3 zt0 represents the time when light is on and zt12 represents the time when light is off). In good agreement with *in vitro* data, we observed a significant increase in phosphorylation of mTORC1 downstream targets at several time points in different tissues of *Bmal1^−/−^* mice: in the cerebellum significant increased activity was observed at zt6 (Figure 3a), in the frontal cortex - at zt2 and zt6 (Figure 3c), in the liver - at zt10 and zt18 (Figure 3b) and in the heart - at zt10 and zt14(Figure 3d). The observed difference in mTORC1 signaling between wild type and *Bmal1−/−* mice can be a consequence of different feeding habits and amount of food consumed by animals: indeed, *Bmal1^−/−^* mice do not display circadian rhythms in gene expression and behavior [31], which can affect their feeding. To exclude this factor of variability, we subjected both wild type and *Bmal1^−/−^* mice to time-restricted feeding (TRF). Mice of both genotypes received the same amount of food (about 95-100% of their daily intake) at the same time. Both groups consumed the food during first three hours after feeding. As shown in Figure 4, although phosphory-lation of S6K1, 4EBP1 and S6 was induced by feeding and gradually reduced with time in both genotypes, the level of phosphorylation in the liver of *Bmal1^−/−^* mice was higher and reduction was considerably delayed compared with wild type. Statistically significant increase in phosphorylation of 4E-BP1 at T37/46 (Figure 4a) and S6K1 at T389 (Figure 4b) was observed at several time points across the daily cycle. This difference cannot arise from variations in the amount of food or feeding behavior, as mice of both genotypes consumed the same amount of food for the same period of time. Taken together, these data suggest that BMAL1 is a negative regulator of mTORC1 signaling *in vitro* and *in vivo*. Importantly, BMAL1-dependent regulation was specific for mTORC1-mediated phosphorylation, because we did not detect increased phosphorylation for the MAPK-specific site T421/S424 of S6K1in the *Bmal1^−/−^* liver (Figure 4c).

### BMAL1 regulates mRNA expression of mtor and deptor

**Figure 5:**
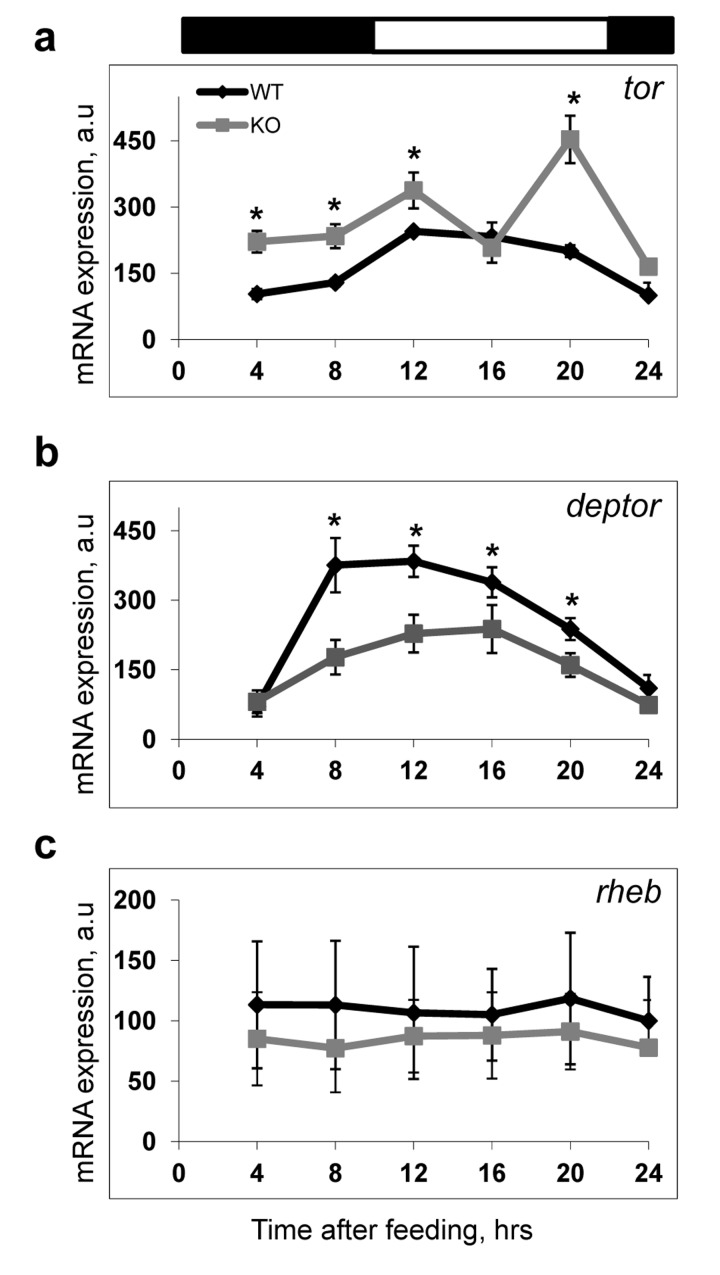
Expression of *mtor* and *deptor* is deregulated in the liver of *Bmal1^−/−^* mice The expressions of several components of TORC1 and upstream regulator of TORC1 in the liver of wild type and *Bmal1^−/−^* mice were analyzed across the daily cycle on mRNAs level by real-time RT PCR. All expression data were normalized to 18S ribosomal RNA expression. **(a-c)** Expression of mRNAs for *tor* **(a)**, *deptor* **(b)**, *rheb* **(c)** in the liver of TRF WT (black diamonds) or *Bmal1^−/−^* (grey squares) mice. Results represent average for 4 animals of each genotype for each time point.* - statistically significant difference between the genotypes. Bars on the top of the figure represent light (open bars) and dark (black bars) parts of the day.

In order to get an insight into possible molecular mechanisms of the BMAL1-dependent regulation of mTOR signaling, we investigated expression of some components of the mTORC1 complex and its upstream regulators. Expression of *tor* and *deptor* mRNA was highly affected by BMAL1 deficiency. Expression of *tor* was significantly upregulated at several time points (Figure 5a), while expression of *deptor* was significantly downregulated (Figure 5b) in the liver of *Bmal1^−/−^* mice in agreement with the increased mTORC1 signaling in these animals. At the same time, we did not detect any significant effect of BMAL1 deficiency on the expression of the mTORC1 upstream regulator *rheb* (Figure 5c), suggesting that the regulation of *tor* and *deptor* expressions is specific. Thus, the BMAL1-dependent regulation may occur at least partially at the transcriptional level, although the existence of other mechanisms cannot be excluded at this point.

### Increased mTOR signaling contributes to accelerated aging of Bmal1^−/−^ mice

Increased mTOR signaling is associated with reduced lifespan, while suppression of this pathway leads to increased longevity in many different organisms [3,4].

**Figure 6:**
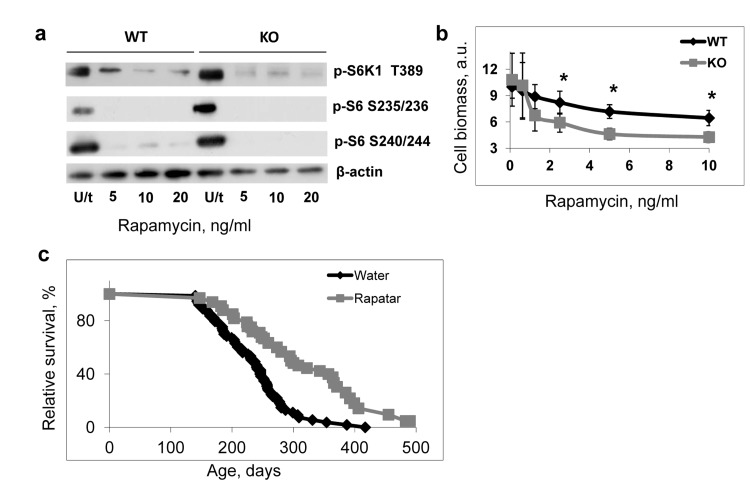
Deregulated mTOR signaling contributes to accelerated aging of *Bmal1^−/−^* mice **(a)** Treatment with rapamycin suppresses TORC1 activity in both wild type and *Bmal1^−/−^* (KO) cells. Cells were treated with indicated concentrations of rapamycin for 4 hrs, protein phosphorylation in cellular extracts were analyzed by western blotting procedure with antibodies recognizing the indicated proteins or protein modifications. U/t untreated cells. **(b)** Effect of rapamycin treatment on proliferation of wild type (black diamonds) and *Bmal1^−/−^* (KO) (grey squares) cells. Cells were grown for 72 hrs in regular growth media with indicated concentrations of rapamycin. Cell biomass was assayed by crystal violet incorporation. Data represent average and standard deviations for 4 replicates. The experiment was repeated 3 times with similar results. a.u. – arbitrary units. * - statistically significant difference between genotypes. **(c)** Kaplan-Meyer survival curves of *Bmal1^−/−^* mice treated with water (N = 73) or Rapatar in drinking water (N = 31). The difference between the survival curves is statistically significant according to logrank test.

Previously we reported that *Bmal1^−/−^* mice developed premature aging and had dramatically reduced lifespan [13]; thus, our current data suggest that the reduced longevity in *Bmal1^−/−^* mice may result from the constitutively elevated mTORC1 signaling. To test this hypothesis we decided to check whether pharmacological suppression of the mTOR signaling by rapamycin (a potent and highly specific inhibitor of mTORC1 kinase activity [32,33]) would increase the lifespan of *Bmal1^−/−^* mice. First we assayed the effect of rapamycin on the BMAL1-dependent regulation of mTORC1 in cell culture. The treatment of cells with rapamycin reduced mTORC1 signaling in both wild type and *Bmal1^−/−^* cells (Figure 6a). *Bmal1^−/−^* fibroblasts were also more sensitive to growth inhibition by rapamycin than the wild type cells (Figure 6b), supporting the role of mTORC1 in the observed difference between wild type and BMAL1-deficient cells.

For *in vivo* administration, we chose to use Rapatar, a polymeric formulation of rapamycin previously characterized by increased bioavailability and distribution [34]. *Bmal1^−/−^* male and female mice received Rapatar in drinking water (approximate cumulative daily dose 0.5mg/kg) throughout their entire lifespan starting at 16 weeks of age. Results of the experiment presented in Figure 6c show the longevity of *Bmal1^−/−^* mice. The median lifespan of untreated mice is 7.8 months and median lifespan of the Rapatar-treated mice is 11.5 months, thus the treatment increased the median lifespan by 50%. The treatment also had a significant effect on the maximum lifespan: the average lifespan of last 25% of survivors for the water control group was 302 days, and for the Rapatar-treated group was 430 days. Therefore, suppression of mTOR activity increases the longevity of *Bmal1^−/−^* mice. This observation supports the hypothesis of the mTORC1-dependent mechanisms of premature aging of these mice, although it is possible that disturbance of several signaling pathways contributes to the accelerated aging phenotype of *Bmal1−/−* mice. Indeed, previously we demonstrated that treatment of *Bmal1^−/−^* mice with antioxidant N-acetyl-cystein also increased their lifespan [35], although the effect was relatively moderate in comparison with the effect of Rapatar.

## DISCUSSION

Activity of mTORC1 is critical for cell growth and proliferation, and disruption of mTORC1 signaling is linked to metabolic diseases, cancer and aging. mTORC1 activity is positively regulated by nutrients and growth factors. Negative regulations of mTORC1 activity by different stresses or sharp nutrient/serum withdrawal have been demonstrated predominantly in cell culture, but little is known about regulation of mTORC1 *in vivo* under physiological conditions. Here we report that the component of the circadian clock transcriptional factor BMAL1 is an important regulator of mTORC1 activity. The circadian clock has been proposed as a master regulator of metabolism. Multiple reports demonstrated clock-dependent regulation of expression and activity of the rate limiting enzymes involved in carbohydrates and fat metabolism [6, 8, 14]. Recently, the circadian clock has been implicated in the control of ribosomal biogenesis and protein synthesis [36]. Our results add an additional layer of circadian control of metabolism through the regulation of mTORC1 activity: indeed, it is well established that through its targets mTORC1 acts as a master switch between cell anabolic and catabolic programs.

We propose that the circadian clock, though BMAL1-dependent mechanisms, inhibits mTOR signaling to actively suppress anabolism and prevent uncontrolled overuse of resources. Anabolic processes such as muscle growth, bone mineralization, hematopoiesis, epithelium and mucous cell renewal, synthesis of hormones and neurotransmitters, building up novel synaptic contacts etc. require significant amount of nutrients and energy; therefore, these processes must be restricted under natural conditions where resources are limited. Indeed, unrestricted growth processes could deplete the pool of available nutrients/energy, leading to disruption of homeostasis and poor fitness, or even rendering the organism unable to sustain the basal metabolic rate; at the same time, excessive growth can prematurely deplete stem cell populations, resulting in accelerated aging. In a sense, uncontrolled growth could also mimic malnutrition (shortage of required nutrients) and also result in poor fitness and premature aging. Thus, the ability of the circadian clock to suppress anabolic processes through mTOR-dependent mechanisms can be evolutionary advantageous and important for survival of mammals in wilderness. Recently, the circadian clock regulation of autophagy through C/EBP dependent mechanisms has been reported [37]; it is possible that this is not the only mechanism. Indeed, suppression of mTOR activity stimulates autophagy [5], therefore, circadian clock regulation of authophagy can also occur through mTORC1-dependent control. Interestingly, in flies the circadian clock was shown to be regulated by the components of the TOR signaling pathway [38, 39]; if a similar regulation exists in mammals, it would suggest the existence of another feedback loop connecting the circadian clock and metabolism.

It is also possible that BMAL1 dependent regulation of TORC1 function is circadian clock independent activity of BMAL1. Indeed, only *Bmal1^−/−^* mice developed accelerated aging phenotype, while other circadian mutants have near normal lifespan [11, 40]. It is necessary to investigate mTOR signaling in the tissues of other circadian mutants to answer the question on circadian clock dependence of the regulation.

In humans, more evidence is accumulated on the importance of the circadian clock in pathology of many diseases. For example, circadian disruption is a recognized risk factor for development of cancer, diabetes and obesity; noteworthy, the mTOR pathway plays an important role in all these diseases. It is also tempting to speculate that disruption of the rhythmic regulation of mTOR signaling can be responsible for the well-known jet leg-initiated dysfunction of the digestive system. The data presented here on the clock-dependent control of mTOR signaling extend our understanding of physiological regulation of the mTOR pathway and provide with potential molecular mechanisms; further studies will result in a more detailed picture of functional interaction between two important regulators of metabolism and aging – the circadian clock and TOR pathway.

## EXPERIMENTAL PROCEDURES

### Animals

*Bmal1^−/−^* mice were obtained from Dr. C. Bradfield (University of Wisconsin), and backcrossed to C57BL/6J inbred strain (The Jackson Laboratory, Bar Harbor, ME, USA) for 12 generations. Wild type and knock out mice were generated by breeding of heterozygous parents. Genotypes were determined using a PCR-based method as previously described [31]. Animals were maintained on a 12:12 light:dark cycle with lights on at 7:00 am, and fed on an 18% protein rodent diet (Harlan). Ad libitum (AL) group had unrestricted access to food. Time restricted feeding (TRF) group received about 100% of their daily intake at zt14, mice have been entrained for TRF for 2 weeks. All groups have unrestricted access to water. All tissue collection experiments have been performed for 3-4 months old wild type and *Bmal1^−/−^* mice. For longevity experiments *Bmal1^−/−^* mice received Rapatar in drinking water at 125mg/L concentration starting 16 weeks of age through the entire life span. Control group received regular water. Water bottles were replaced twice/week. Polymeric formulation of rapamycin (Rapatar) was developed by Tartis Aging, Inc. using Pluronic block co-polymers [41] as previously described [34]. All animal studies were conducted in accordance with the regulations of the Committee on Animal Care and Use at Cleveland State University and Roswell Park Cancer Institute.

### Analysis of protein phosphorylation and expression

Tissue and cell extracts were normalized by measuring total protein concentration using Bio-Rad Dc protein Assay kit according manufacturer's protocol. Extracts were separated by SDS-PAGE, transferred on Immobilon-P membrane (Millipore) and incubated with phosphospecific and protein specific primary and secondary antibodies (Cell Signaling and Santa Cruz Biotechnoly). Due to technical difficulties, instead of stripping and re-probing the same membrane with different antibodies, several gels loaded with the same extracts were run in parallel; after staining with Ponceau S Red and visual examination of the transfer quality, one membrane was stained with phosphor-specific antibodies and another with antibodies specific for the total protein. Experiments with the same extracts were repeated independently at least two times. Following antibodies were used: Cell Signaling Technology: Phospho-p70 S6K (Thr389) #9205, Phospho-p70 S6K (T421/S424) #9204, p70 S6K #9202, Phospho-4E-BP1 (T37/46) #2855, 4E-BP1 #9452, Phospho-S6 (Ser235/236) 32211, Phospho-S6 (Ser240/244) #2215, Anti-Mouse IgG-HRP #7076, aAnti-rabbit IgG-HRP #7074. Santa Cruz Biotechnology: Robosomal Protein S6 sc-74459, Donkey anti-rabbit IgG-HRP sc-2313. Abcam: Anti-S6K1 (phosphor T389) ab2571.

### Analysis of mRNA expression

Total RNA was isolated with TriZol reagent (Invitrogen) according with the manufacturer's protocol. RNA quantization was performed using real-time RT-PCR with Syber Green mix (BioRad), relative mRNA abundance was calculated using the comparative delta-Ct method with 18S RNA as standard as described in [42]. Following primers have been used for the analysis of expression:

18s rRNA F 5' GCT TAA TTT GAC TCA ACA CGG GA 3'
18s rRNA R 5' AGC TAT CAA TCT GTC AAT CCT GTC 3'
MTOR F 5' ATT CAA TCC ATA GCC CCG TC 3'
MTOR R 5' TGC ATC ACT CGT TCA TCC TG 3'
DEPTOR F 5' GTG GTT CTC AGG CAT TCT ATC TC 3'
DEPTOR R 5' TGG GTA GGT TTT GAG ATG GTG 3'
RHEB F 5' AAG ATG CCT CAG TCC AAG TC 3'
RHEB R 5' CGT GTT CTC TAT GGT TGG ATC G 3'

### Cell culture

All cells were cultured in Dulbecco's modified Eagle's medium (LRI, Cleveland Clinic, Cleveland, USA) supplemented with 10 % fetal bovine serum premium (ATLANTA Biologicals, USA) and 10000 units Of Penicillin G and 10000 μg/ml streptomycin (LRI,OH,USA). Cells were maintained at 37°C in humid atmosphere with 5 % CO_2_ in air. To generate primary lung fibroblasts lungs isolated from WT AND Bmal1 KO mice were cut to small pieces and placed in cell growth media. After 72 hrs media were replaced and all unattached cells were removed. For serum starvation or amino acids starvation experiments cells were maintained in the media without serum or without amino acids. Cell size was analyzed by using FACSCalibur cell sorter (Becton-Dikinson). To assay cell protein contents cells were counted, equal number of cells was dissolved in fixed volume of RIPA buffer, protein concentration in extracts was analyzed using Bio-Rad Dc protein Assay kit according manufacturer's protocol. For rapamycin sensitivity assay. WT and *Bmal1 −/−* cells were plated in 96 well plates at a density of 1 × 10^3^ cells per well. 24 hrs after plating cells were treated with different concentrations of rapamycin (Sigma- Aldrich, MO,USA) for 24, 48 and 72 hrs. Cells proliferation was estimated using two independent protocols. MTT assay, incorporation and detection of MTT reagent (Sigma- Aldrich, MO,USA) was performed according to the manufacturer protocol. In independent set of experiments treated cells were fixed with 0.2 % glutaraldehyde (Fischer Scientific,USA) and stained with 0.05 % crystal violet solution. Incorporated dye was extracted with 1% SDS solution and amount was estimated as optical density at 570 nM using Victor^3^ 1420 plate reader.

### Statistical analysis

At least 4 animals for every time point, for both feeding types and for each genotype were used for all experiments. Data are shown as mean + standard deviation. SigmaStat software package was used for analysis. Effects of genotype (circadian mutants versus wild type), feeding type (AL versus TRF) and time of the day were tested for significance. Unpaired Student's t-test was used for comparison. 31 *Bmal1−/−* mice (both genders) were treated with rapatar and 73 *Bmal1−/−* mice (both genders) treated with control solution were used for the longevity experiment.. If mice need to be sacrificed according veterinarian recommendation due to morbidity or some severe pathologies, these mice were counted as alive before the day of sacrifice and as missing after the day of the sacrifice. Because the treatment was started with mice 16 weeks of age (102 days), we excluded from the consideration all animals which have died before the beginning of the treatment or animals which have died during first month of treatment in both control and treated groups. Log-rank test was used for analysis of longevity experiments. P<0.05 was considered as statistically significant difference.
